# Hereditary chromosomal 9 inversion (p22q13) 9 as a cause for recurrent pregnancy loss: a case report

**DOI:** 10.1186/s13256-023-04137-z

**Published:** 2023-10-13

**Authors:** Mohammad Marwan Alhalabi, Ameer Kakaje, Marwan Alhalabi

**Affiliations:** 1 University of Damascus Faculty of Medicine, Damascus, Syria; 2 Department of Reproductive Medicine, Genetics and Embryology, Damascus University, Damascus, Syria; 3 BioTherapeutics Research Center, Damascus University, Damascus, Syria

**Keywords:** Chromosome, Genes, Infertility, Inversion, Pregnancy loss

## Abstract

**Background:**

Chromosomal aberrations are as common as 13.8% in the infertile population. The incidence of pericentric inversion of chromosome 9 is approximately 1–3%. However, although these inversions do not alternate phenotype, there have been conflicting data about their effect as they were correlated with infertility, recurrent pregnancy loss, and deceased children, with no clear evidence of the inversions being the causative factor for these events.

**Case presentation:**

We report a case report of an Arab family with many members with inv(9)(p22q13). Our proband male aged 35 years at time of presentation with primary infertility. Some members, such as a brother aged 34 years, who had this inversion suffered from recurrent pregnancy loss while other members of similar reproductive age did not.

**Conclusions:**

inv(9)(p22q13) might be a hereditary anomaly that might be a risk factor for recurrent pregnancy loss in its members.

## Introduction

As much as 15% of infertility problems are caused by chromosomal anomalies [1], with the 9th chromosome having the highest degree of variability in structure [2]. Different breakpoints of chromosome 9 inversions can cause diverse disruptions in the carriers. Pericentric inversions occur when a double breakage occurs in each arm of an affected chromosome, which includes the centromere [3]. Although they are dominant in characters that affect both sexes equally, they rarely lead to phenotypic abnormality, and they are of an occurrence of 50% to be inherited if the parent was heterozygous. Having the inverted regions enclose nothing but the chromosomal centromere and its heterochromatin, it rarely causes any genetic aberrancy after crossing over. It was also reported that structural rearrangements of chromosomes could be inherited like dominant characteristics, leading to hypothesized phenotypic abnormality [4, 5]. Moreover, many cytogeneticists consider pericentric chromosome 9 inversions in the heterochromatin as a normal variance, especially when there is no corresponding phenotype. Such is the case of the relatively common pericentric inversion inv(9) (p11q12) and inv(9)(p11q13) [6]. In the general population, it is said that the incidence of which is about 1–3% [7]. However, regardless of being a minor chromosomal rearrangement not related to any alterations in the phenotype, it was reported in the literature with some conflicting data. It was associated with some medical conditions such as infertility, recurrent pregnancy loss, and even deceased infants. Concluding an unclarity on whether inv(9)(p11q12) should be considered normal or abnormal [8]. Heterochromatin variance was also reported to probably cause instability in the chromosome, cancer, and even congenital abnormalities. In addition, carriers of such genetic abnormality are at risk of developing abnormal gametes during meiosis resulting in unbalanced offspring [6, 9, 10]. The phenotype of heterozygosity of this inversion ranges from being normal to a wide range of abnormalities [11]. Furthermore, the chromosomal abnormality inv(9)(p11q13) mentioned as normal earlier was also noted to cause skull and facial abnormalities, mental and growth retardation, congenital skeletal and cardiac malformation, and habitual aborts [12–14]. Infertility is defined as the inability of a couple to conceive after 12 months of regular unprotected sexual intercourse [15]. The World Health Organization (WHO) defines the term primary infertility as when a couple has never achieved conception, and the term secondary is the inability to conceive for a couple who had at least one conception [16]. Recurrent pregnancy loss is defined as two or more pregnancy losses according to the American Society for Reproductive Medicine (ASRM) [17]. Our study presents cases of multiple familial obstetric medical outcomes affecting a whole family and suspected to be resulting from a hereditary 9th chromosomal abnormality.

## Case

A nonconsan­guineous proband couple with Syrian ethnicity presented to the infertility clinic with the complaint of primary infertility lasting for 7 years. The husband who was the proband for his family did not complain of any symptoms affecting ejaculation or sexual intercourse. He had a normal clinical examination, with both testes appearing normal in size and shape. The patient's wife had a history of mononeuritis multiplex, and the proband husband had no medical history but had a history of surgery for a bilateral varicocele of the spermatic cord. The patient was referred to conduct semen analysis which was within the normal ranges for each feature tested. They had no history of drug intake, or exposure to radiation or chemicals. Transvaginal ultrasonography showed a normal uterus with a normal antral follicle count; around 11 in each ovary (hyporesponder < 5 follicles, suboptimal responder 6–10, optimal 11–15, hyperresponder > 15), and the size of the follicles ranged from 5 to 10 mm (follicles were measured on day 3 of the cycles and only the follicles ≤ 10 are counted). Basal hormones on day 3 of cycle were normal [FSH 6.7 mIU/ml (normal range 3.4–10 mIU/ml) LH 4.6 mIU/ml (normal range 1.6–8.3 mIU/ml) Prolactin 13 ng/ml (normal range 3.6–20 ng/ml) Testosterone 0.6 nmol/l (normal range 0.5–2.5 nmol/l) Oestradiol 34 pg/ml (normal range < 50 pg/ml) Progesterone 0.4 ng/ml (normal range 0.32–2 ng/ml). hysterosalpingogram showed normal fallopian tubes. The wife also had some anovulatory cycles, so ovulation induction was tried with clomiphene citrate as simple treatment. Despite the successful ovulatory cycles and successful intrauterine inseminations with the addition of gonadotropins, she failed to conceive. The proband couple was later referred for in-vitro fertilisation (IVF) due to the unexplained infertility.

The husband's brother later on referred to the clinic complaining of recurrent pregnancy loss of four consecutive pregnancies over the course of 5 years. This couple did not have any medical history, and the surgical history was also unremarkable, but the husbands' family had a similar history of four spontaneous abortions for the husbands' parents. Physical examination of the reproductive system for the brother was normal. The brother’s seminogram was also within normal limits according to WHO 2010 criteria. His wife underwent a hysterosalpingogram which was also unremarkable. She also underwent immuno-testing and was lab tested for Anticardiolipin IgG and IgM, ANA, Lupus anticoagulant, Homocysteine, Antiphospholipid IgG and IgM, Thyroid peroxidase, Protein C resistance, TNF-alpha, and all were within normal ranges. Genetic causes were suspected and both couples were referred for karyotyping of peripheral blood cells. The karyotype was tested using the G-Banding technique [18], and all results were reported according to the standard of the 2016 protocols of the International System for Human Cytogenetic Nomenclature (ISCN) [19].

**Fig. 1:**
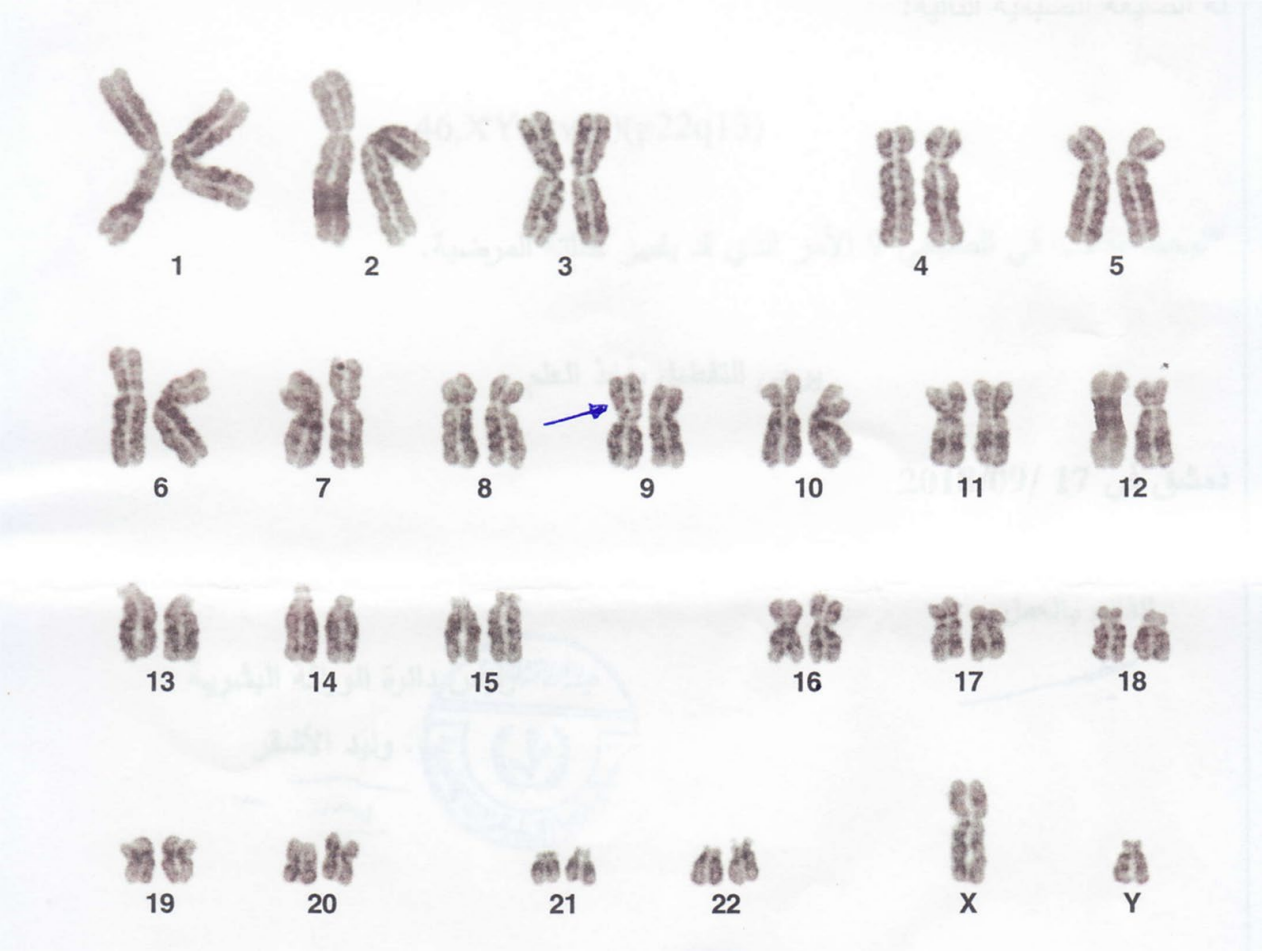
Karyotype test result of the proband. Blue Arrow: heterozygous chromosomal rearrangement of pericentric chromosome 9 inversion (p22q13)

**Fig. 2:**
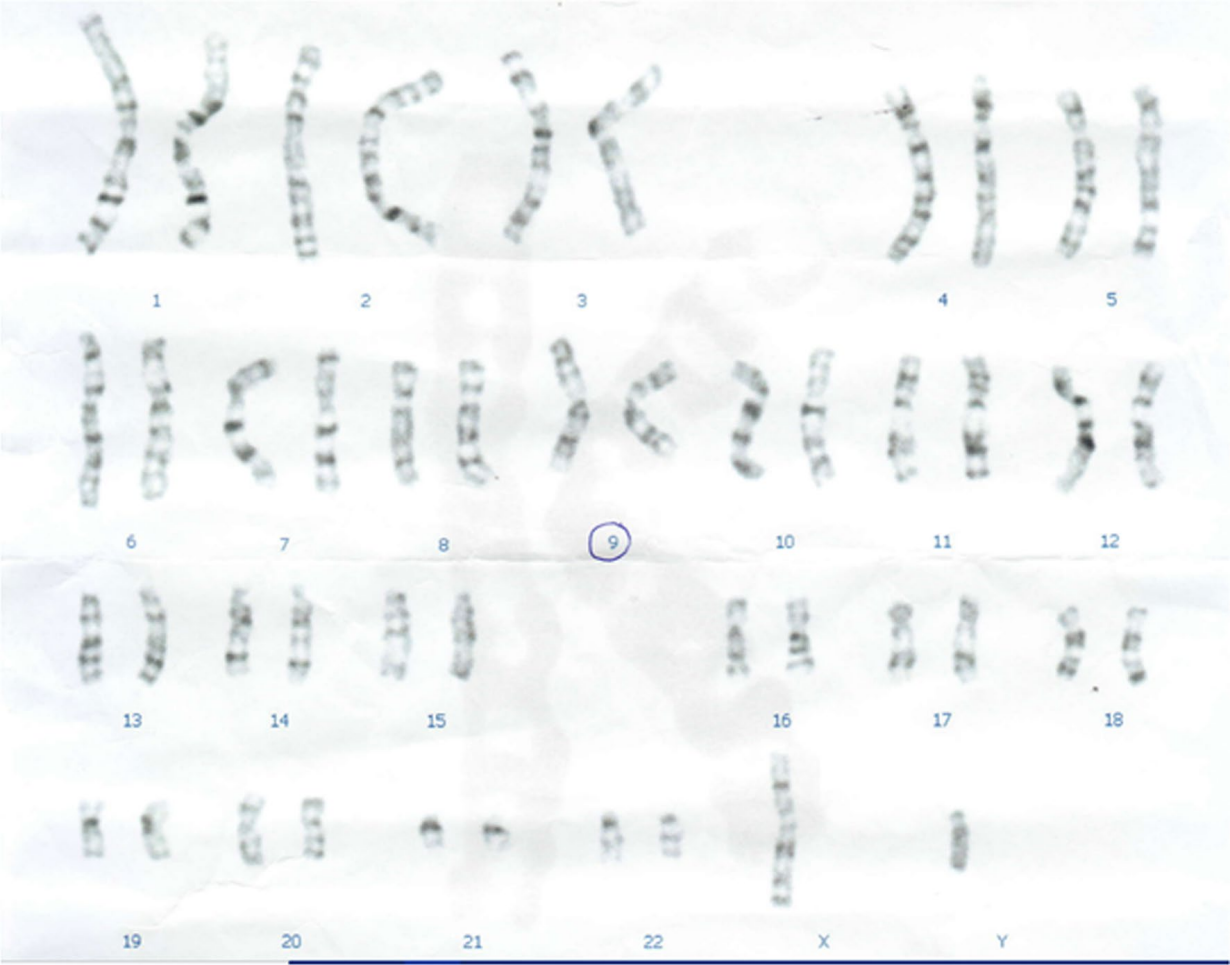
Karyotype test of the proband's brother with an arrow indicating the homozygous mutation 46,XY,inv(9)(p22q13)x2

Both the husbands’ karyotype tests showed a common finding of 46,XY,inv(9)(p22q13), but the proband’s brother’s karyotype had a homozygous chromosomal variation, namely 46,XY,inv(9)(p22q13) × 2 (Fig. 1 and 2). Wives karyotyping results were normal for 46,XX.

**Fig. 3:**
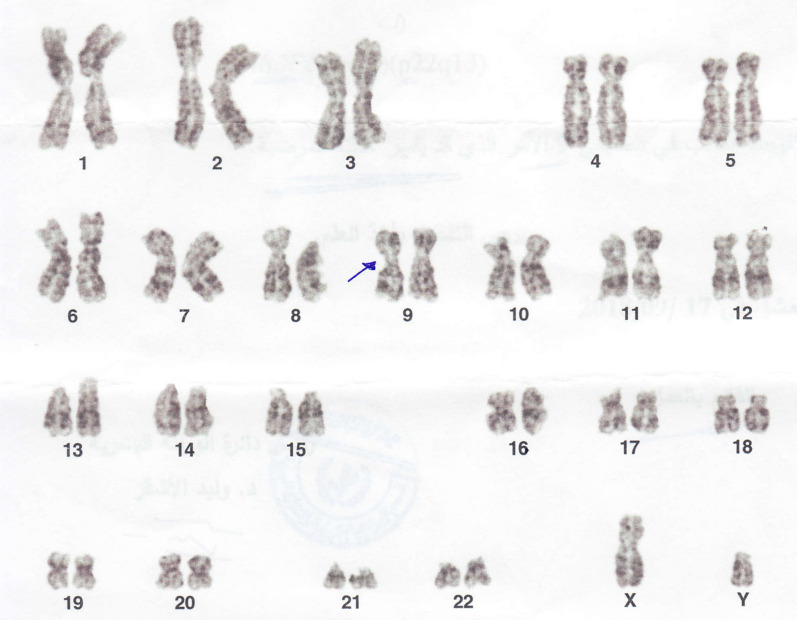
Karyotype test result of the family's mother. Blue Arrow: heterozygous chromosomal rearrangement of pericentric chromosome 9 inversion (p22q13)

**Fig. 4:**
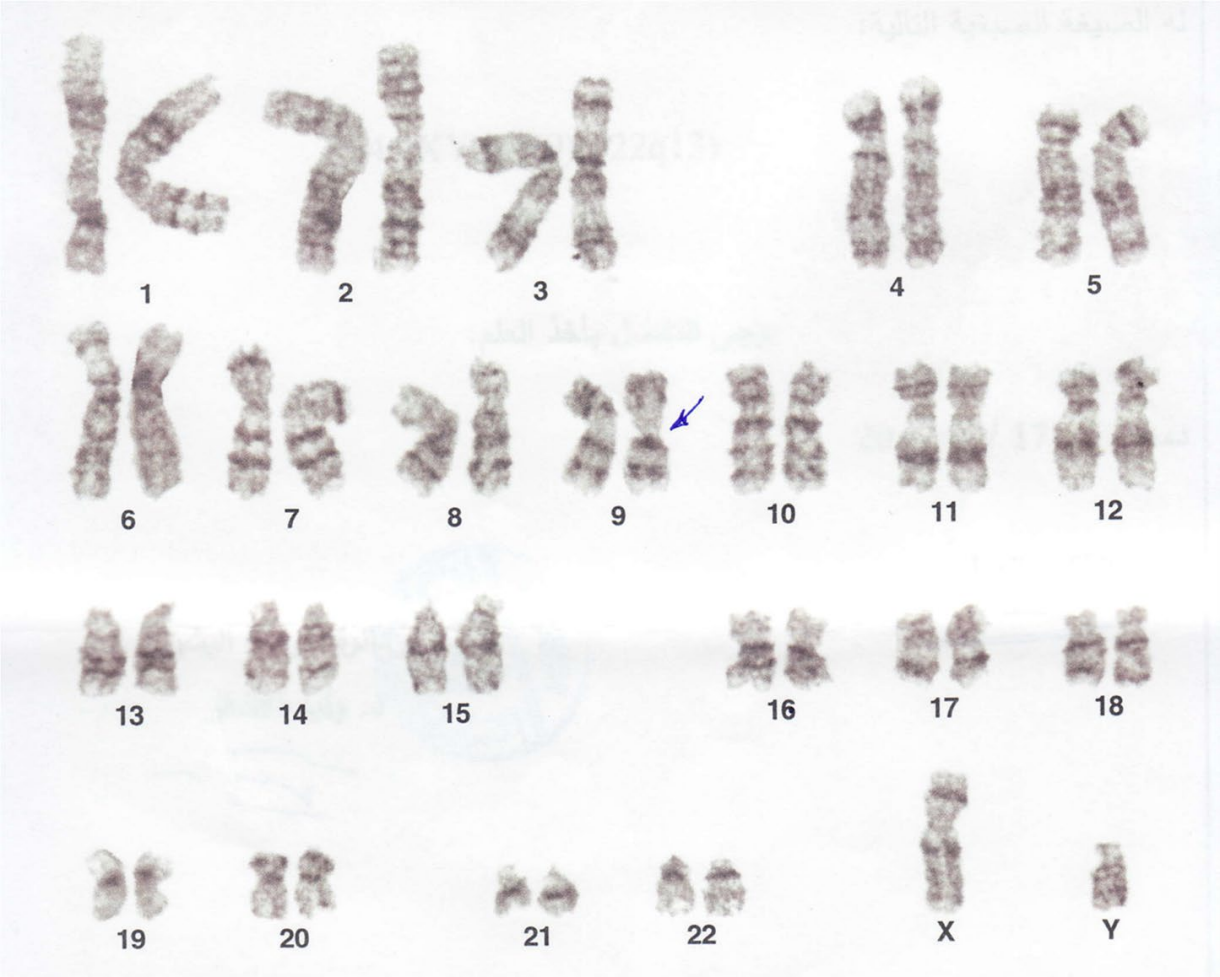
Karyotype test result of the proband's second brother. Blue Arrow: heterozygous chromosomal rearrangement of pericentric chromosome 9 inversion (p22q13)

**Fig. 5:**
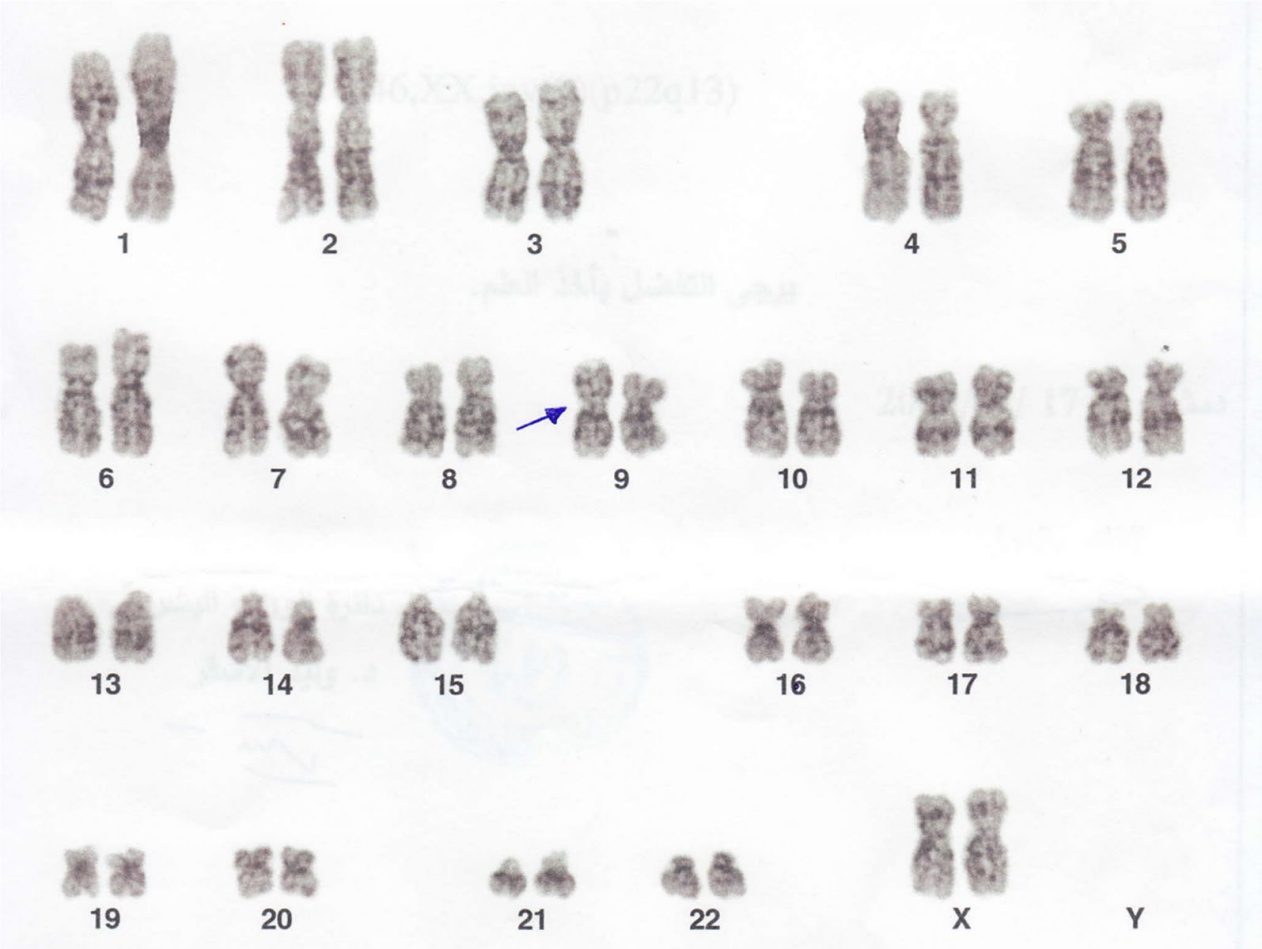
Karyotype test result of the proband's sister. Blue Arrow: heterozygous chromosomal rearrangement of pericentric chromosome 9 inversion (p22q13)

**Fig. 6:**
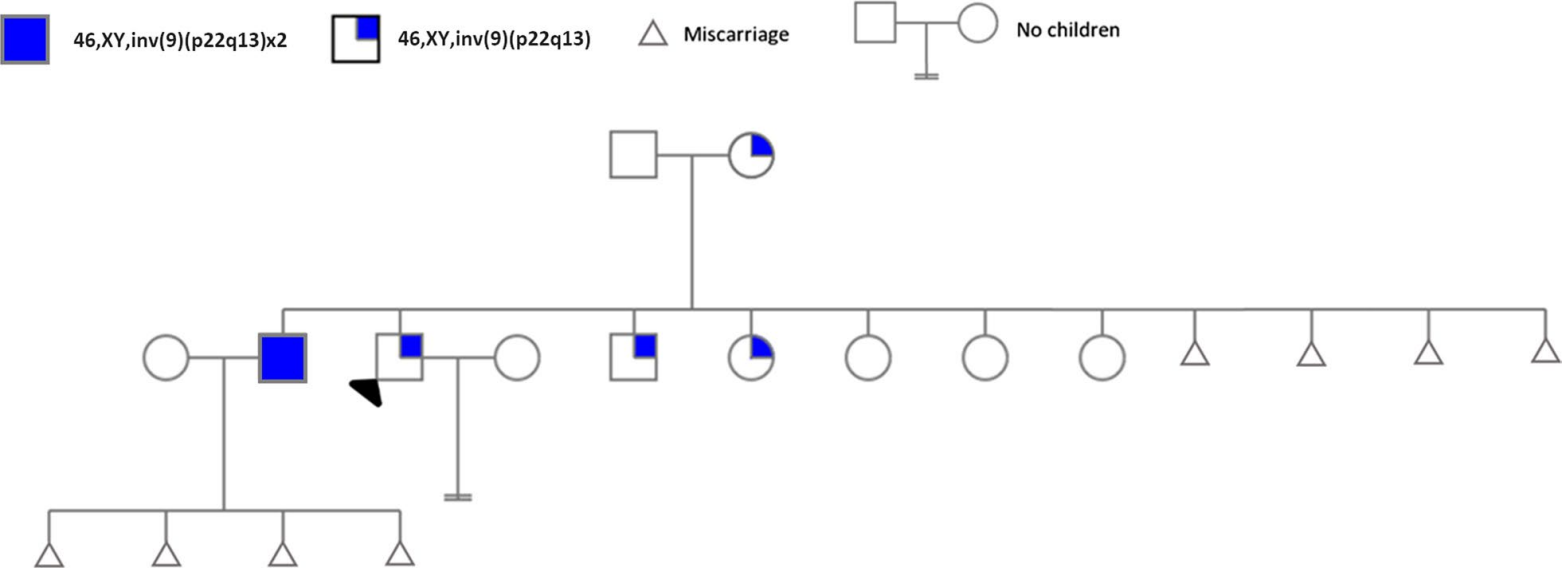
Family pedigree of the chromosomal aberration

However, the wives were normal (46,XX). The patients’ families were referred for genetic testing, and the results were that one parent of both affected husbands, that is, the mother, also was a carrier of the genetic abnormality (Fig. 3). Additionally, it was affecting a brother and a sister of both the husbands (Figs. 4 and 5), all sharing the same genetic abnormality of the ninth chromosome of inv(9)(p22q13) (Fig. 6).

## Discussion

Familial pericentric inversion of chromosome 9 (p22q33) was described as early as 1980 to cause a malformed child, and it was found that even second-degree cousins had the same clinical features [12]. This emphasises the importance of the familial study when such an anomaly is found. It was believed that chromosomal inversions are rarely detected by using traditional staining. However, using band techniques, which allow a more precise method of detecting such anomalies, minor pericentric inversions were found to be more common than initially found [20, 21].

It was found that children born from a heterozygous mother are more than those born from a heterozygous father, which may suggest a relatively declined fertility in males compared to females, or a relatively improved fertility in females compared to males [22]. Although a study found that chromosome 9 inversions were more common in females, especially in those who suffer from infertility [23], Inv(9) was studied in the male sperm, and it was found that the DNA in the sperm had many more abnormalities such as fragmentations, alterations in the meiosis, aneuploidy, and changes in seminogram parameters [9]. This concurs with our study in that we found the proband couple’s infertility to be of male factor.

Furthermore, it was found in one study that all inverted chromosomes were identical within the family of the pedigree, having the same length in the normal homologous with the result that there is no loss of material. There was no evidence of new mutations in individuals coming from two such parents. Therefore, there were no de novo chromosomal inversions [24]. This could explain our finding of the same chromosomal aberration in all of our studied pedigree.

Chromosomal inversions might be even more common than anticipated because tests that detect them are only requested on clinical grounds, neglecting the subclinical presentations or cases that cause no phenotypic abnormality whatsoever [5, 25]. Such is the case in how our studied pedigree’s genetic abnormality was detected based on the symptomatic presentation in our clinic. Most patients appear normal and relatively can reproduce; hence, inversion carriers may propagate such chromosomal aberrations without even noticing any abnormalities [11].

It is also difficult to conclude whether any of the inversions truly increases spontaneous abortions. One family whose mother had heterozygous inv(9) suffered from four spontaneous abortions with no children. The first and second metaphases appeared to be normal, which may suggest that the meiotic division was not grossly affected, though it does not rule out the effect of crossing over [24]. Accordingly, chromosomal aberrations could be responsible for as much as 50% of all spontaneous abortions and miscarriages, in addition to many congenital abnormalities [10, 26]. Many reports are emerging in the medical literature on the importance of chromosome 9 inversions in correlation with unfavourable obstetric history [27, 28]. Correspondingly, chromosome 9 inversion was reported in 2.3% of couples who suffered from recurrent spontaneous miscarriages and infertility [29]. One of our patients, namely the proband’s brother, and the family’s parents, also suffered from four spontaneous abortions. The brother’s abnormality was homozygous, and no other cause of spontaneous abortion was found. Also, we can only assume that the patient's brother with the homozygous variation might have suffered from heterodisomy in inheriting two copies from the heterozygous mother, similar to one case in a previously reported study [30]. Furthermore, the proband and his brother also had seminograms within normal limits, and despite the proband's surgical history, this did not affect the semen quality.

However, we cannot know for certain whether to count inv(9) as normal or abnormal [8] because it sometimes raises the instability of the chromosome, malignancies, and congenital malformations [12–14]. Chromosome 9 has the highest morphological variance, with inversions being the most common alteration, and it is believed that it is being inherited by Mendelian rules [8, 31]. Moreover, such a common abnormality was reported in a few studies to be related to infertility and congenital malformations [32]. Therefore, it is believed it should be considered harmful, especially with the aforementioned reports suggesting its negative effect on obstetric history, sterility, and frequent miscarriages [27, 28, 33, 34]. In contrast, other reports reported a correlation of such findings with abnormalities in chromosomes 1 and 10 [4, 9, 35]. However, our study was in line with the inherited chromosome 9 abnormality being the causative factor.

Phenotypes of Inv(9) depend on the breakpoint locations, for example, (p11q12), which is believed to have one type of breakpoint at (p11q12), with the result that it leads to no effect on the carrier nor causes any imbalances or miscarriages in the pedigree [36]. Moreover, during the reunion after the breakage, many alterations may occur in the euchromatic sequences ranging from suppressing to deleting, which can cause various abnormalities [37]. Such can explain the persistence of alterations in one pedigree. It was also reported that chromosome 9 [46,XX,inv(9)(p11-q13)] could phenotype as complete hydatidiform mole [38], and inv(9) can also predispose to schizophrenia [36]. Different breakpoint regions for inv(9) correlated with different clinical aspects. More studies using molecular cytogenetic probes on a genome-wide basis are needed in order to make causative relations between each type of chromosomal 9 inversion and their distinct clinical syndromes.

In Conclusion, inv(9)(p22q13) can be a hereditary chromosomal anomaly which might be a risk factor for recurrent pregnancy loss, though literature data might be conflicting on the matter. Cytogenetic investigation could be a mainstay diagnostic step in the standard of care for cases of unexplained recurrent pregnancy loss, in addition to unexplained infertility, especially in patient populations sharing a similar familial obstetric history.

## Data Availability

The data can be made available upon reasonable request.

## Abbreviations

ANA: Antinuclear Antibody
ASRM: American Society for Reproductive Medicine
FSH: Follicle Stimulating Hormone
Ig: Immunoglobulin
Inv: Inversion
ISCN: International system for human cytogenetic nomenclature
IVF: In-vitro fertilisation
LH: Luteinizing Hormone
TNF: Tumour necrosis factor
TNF-alpha: Tumor Necrosis Factor alpha
WHO: World Health Organization
